# Time-resolved x-ray/optical pump-probe simulations on N_2_ molecules

**DOI:** 10.1063/1.5053995

**Published:** 2019-03-18

**Authors:** Athiya Mahmud Hanna, Oriol Vendrell, Robin Santra

**Affiliations:** 1 Center for Free-Electron Laser Science, DESY, Notkestraße 85, D-22607 Hamburg, Germany; 2 Department of Chemistry, Universität Hamburg, Martin-Luther-King-Platz 6, 20146 Hamburg, Germany; 3 Hamburg Centre for Ultrafast Imaging, Luruper Chaussee 149, D-22761 Hamburg, Germany; 4 Theoretische Chemie, Physikalisch-Chemisches Institut, Universität Heidelberg, Im Neuenheimer Feld 229, 69120 Heidelberg, Germany; 5 Department of Physics, Universität Hamburg, Jungiusstraße 9, D-20355 Hamburg, Germany

## Abstract

The dynamics of ${N_{2}}^{2+}$ dications after x-ray-induced Auger decay and their probing with a delayed infrared probe pulse are theoretically investigated based on a quantum-mechanical model including all relevant electronic states for which wave-packet calculations on *ab-initio* potential energy curves are performed. Our results demonstrate that the ${N_{2}}^{2+}$ yield modulated by the delay of the probe pulse contains dynamical information on the wave-packet evolution in the quasi-bound final electronic states. The Fourier transform of the calculated yield can be readily compared to experimental results [Fung *et al.*, Nature **532**, 471 (2016)] and good agreement of the main frequencies is found. Moreover, assignment of these frequencies to specific vibrational energy levels in the quasi-bound potentials is reported as well.

## I. INTRODUCTION

The dynamics associated with the Auger decay of nitrogen molecules has been the subject of recent studies based on ultrafast x-ray sources.^1–5^ In fact, N_2_ was the subject of the first time-resolved x-ray pump–IR probe experiments at the Linac Coherent Light Source (LCLS).^1^ After removal of a core electron via electron impact ionization or photoionization, N_2_ decays primarily into dicationic nitrogen molecules ${N_{2}}^{2+}$.^6^ The molecular dications may end up as metastable ${N_{2}}^{2+}$ in quasi-bound electronic states featuring a barrier towards molecular fragmentation or undergo further symmetric (${N_{2}}^{2+}\to N^{+}+N^{+}$) or asymmetric fragmentation (${N_{2}}^{2+}\to N+N^{2+}$).^5,6^ Many studies on the Auger process in nitrogen molecules have been devoted to uncover these fragmentation dynamics focusing on Auger spectra,^7–12^ the core-hole states from which the Auger process begins,^2,11,13–15^ and the characterization of the quasi-bound and dissociative dicationic states populated by the Auger decay.^1,5,16^ Most of the available works are based on measurements of properties of the fragmentation products, e.g., the kinetic energy release of the fragments or Auger electron energies, while the real-time nuclear dynamics triggered by x-ray absorption followed by Auger decay remains comparatively much less explored.

A time-resolved experimental study on the dynamics after the Auger process was conducted by Glownia *et al.*^1^ using an x-ray pump–IR probe setup at LCLS. They found an enhancement of ${N_{2}}^{2+}$ dissociation as a function of the pump-probe delay when the IR pulse was set up to operate after the x-ray pulse. Unfortunately, the timing uncertainty of a few hundred femtoseconds of the delay between both pulses^1^ rendered more precise analysis of the data extremely challenging. Later on, those experimental data were re-analyzed based on a newly developed singular-value-decomposition scheme where the timing uncertainty of the original data could be largely compensated.^4^ A recent experimental and theoretical study was carried out to investigate the x-ray-induced wave-packet dynamics of ${N_{2}}^{2+}$ after Auger decay.^5^ In this study, the kinetic-energy release of the ion fragments was measured. It was found to decrease as a function of the pump-probe delay time signaling the progress of dissociation in dissociative channels, whereas it was observed to remain constant for the quasi-bound channels of ${N_{2}}^{2+}$. A separate experimental study by Iwayama *et al.*^17^ employed coincidence measurements of the Auger electron and the ions following the Auger decay and indicated that the metastable states featuring quasi-bound potential energy curves and leading to ${N_{2}}^{2+}$ formation are found for binding energies up to about 50 eV, in agreement with the window of final states considered in our previous theoretical study^16^ and in this work.

The quasi-bound states of ${N_{2}}^{2+}$ and the vibrational dynamics on their potential energy curves have been, on their own, the subject of numerous theoretical and experimental investigations.^18–25^ Early theoretical studies of ${N_{2}}^{2+}$ by Wetmore and Boyd provided potential energy curves of the dissociative and quasi-bound states and discussed the tunneling dissociation properties of the latter.^18^ The theoretical study of Taylor and Patridge concluded definitively that the ground state of the ${N_{2}}^{2+}$ system is $X^{1}\Sigma_{g}^{+}$, ending a previously unresolved dispute.^19^ A recent theoretical investigation by Fantuzzi *et al.* focused on the electronic-structure origin of quasi-bound potential energy curves in homonuclear dicationic molecules including ${N_{2}}^{2+}$ and found that the local minima of the dication states arise from a strong stabilization by polarization-aided interference effects.^25^ The spectroscopic properties of vibrational states of quasi-bound potential energy curves in ${N_{2}}^{2+}$ were the subject of theoretical and experimental investigations,^20–22^ and a theoretical study by Jiang *et al.* used a Floquet picture to study the wave-packet dynamics in the quasi-bound potentials and their dissociation in the presence of a strong laser field.^23^

In this study, the possibility to obtain dynamical information on the vibrational wave packets of ${N_{2}}^{2+}$ after Auger decay through modulations in the yield of decay products in an x-ray pump–IR probe setup is investigated theoretically. We introduce a model that includes the relevant initial, intermediate, and final electronic states and the pump and probe pulses. The nuclear and electronic dynamics in this model are described quantum mechanically in a time-dependent picture. Based on this model, we calculate the variation of the ${N_{2}}^{2+}$ yield as a function of the pump-probe delay time and show that the Fourier transform of this time-signal yields information on the vibrational wave packets created after the Auger decay process in the quasi-bound potential energy curves. Since the real-time x-ray-pump–IR-probe simulations are computationally expensive (cf. Sec. II A), a perturbative treatment of the probe pulse is introduced and described as well. Our results can be directly compared to the study by Fung *et al.*,^4^ where the delay-dependent ${N_{2}}^{2+}$ yield obtained experimentally^1^ was analyzed in detail. The potential energy curves and transition dipole moments used in this study, as well as the quantum-mechanical model of the pump and probe steps, have been described in detail in Ref. 16.

This paper is organized as follows. The theoretical and computational methods for full pump-probe simulations and the perturbative analysis are described in Secs. II A and II B, respectively. The results from the perturbative model are then discussed in Sec. III A, and the results from the pump-probe simulations are discussed in Sec. III B. Finally, the work is summarized in Sec. IV.

## II. THEORETICAL AND COMPUTATIONAL DETAILS

The theoretical model used to describe the Auger decay of ${N_{2}}^{+}$ and the subsequent wave-packet dynamics of the final states was presented elsewhere,^16^ including a detailed description of the potential energy surfaces, transition dipole moments, and decay width parameters. The relevant set of differential equations in atomic units is given here for the sake of completeness

$$i\frac{\partial }{\partial t}|\Phi_{i}(t)\rangle ={\hat{H}}_{i}|\Phi_{i}(t)\rangle ,$$ (1)

$$i\frac{\partial }{\partial t}|\Phi_{d}(t)\rangle ={\hat{A}}_{x}(t)|\Phi_{i}(t)\rangle +\left({\hat{H}}_{d}-i\frac{\hat{\Gamma }}{2}\right)|\Phi_{d}(t)\rangle ,$$ (2)

$$\begin{array}{c} i\frac{\partial }{\partial t}|\Phi_{f}(E,t;\tau )\rangle ={\hat{W}}_{f}|\Phi_{d}(t)\rangle +({\hat{H}}_{f}+E)|\Phi_{f}(E,t;\tau )\rangle \\ +\sum_{g\neq f}{\hat{\mu }}_{\mathrm{fg}}\cdot e_{\text{IR}}\;\varepsilon (t-\tau )|\Phi_{g}(E,t;\tau )\rangle . \end{array}$$ (3)

In Eqs. (1)–(3), $\left|\Phi_{i}(t)\rangle ,\;|\Phi_{d}(t)\right\rangle$, and $\left|\Phi_{f}(E,t;\tau )\right\rangle$ are time-dependent nuclear wave functions of the initial, intermediate, and final states, respectively, and *E* is the Auger electron energy. Note that the wave functions of the final states are Auger-energy-dependent,^16^ which is a consequence of total energy conservation during the Auger decay process, and they parametrically depend on the delay time of the probe pulse, *τ*. ${\hat{H}}_{\left(i/d/f\right)}={\hat{T}}_{\text{nuc}}+{\hat{V}}_{\left(i/d/f\right)}$ is the nuclear Hamiltonian of the initial, intermediate, and final states, respectively, where ${\hat{T}}_{\text{nuc}}$ is the kinetic energy of the nuclei and ${\hat{V}}_{\left(i/d/f\right)}$ is the potential energy curve of the corresponding electronic states. ${\hat{A}}_{x}(t),\;\hat{\Gamma },\;{\hat{W}}_{f}$, and ${\hat{\mu }}_{\mathrm{fg}}$ are the x-ray pulse envelope, the energy decay width of the intermediate state, the transition operators between the intermediate and the final states, and the transition dipole moment operators between final states *f* and *g*, respectively. *ε*(*t* − *τ*) is the electric field amplitude of the IR probe pulse centered at delay time *τ* and **e**_IR_ indicates its polarization direction. The potential energy curves of the final dicationic states considered in this study consist of the quasi-bound electronic states $X^{1}\Sigma_{g}^{+},\;2^{1}\Sigma_{g}^{+},\;1^{1}\Pi_{u}$, and $1^{1}\Sigma_{u}^{+}$ and the dissociative states $1^{1}\Delta_{g},\;1^{1}\Pi_{g},\;2^{1}\Pi_{g}$, and 3^1^Π_*g.*_^16^ One core-ionized intermediate state is considered in the model.^16^

The nuclear wave functions are described using two degrees of freedom, namely, the interatomic distance *R* and the angle *θ* between the molecular axis and the IR polarization direction.^16^ The wave-packet propagations were performed with the multi-configuration time-dependent Hartree method using the Heidelberg package.^26^ The constant mean-field integrator^27–29^ was used with an initial stepsize of 0.05 fs and an error threshold of 10^−9^. This gave an accurate propagation significantly faster in comparison to the more accurate variable mean-field propagator used in our previous work.^16^ The wave-packet propagations were carried out using shared-memory parallelization on 12-core Intel^®^ Xeon^®^ X5650 CPUs with 2.67 GHz and 96 GB of memory.

### A. Pump-probe simulations

In this work, we are interested in the dynamical information that can be extracted from metastable ${N_{2}}^{2+}$ after core-shell ionization and subsequent Auger decay, as a function of the delay time of an IR probe pulse. The IR pulse interacts with the system after the Auger decay and modifies the amount of unfragmented ${N_{2}}^{2+}$ that reaches the detector. The yield of ${N_{2}}^{2+}$ reaching the detector as a function of the pump-probe delay thus contains information on the metastable vibrational wave packets created after the Auger decay and is defined as

$$\Omega (\tau )=\sum_{f}\int dE\;\langle \Phi_{f}(E,t_{D};\tau )|\hat{\Theta }(R_{b}-R)|\Phi_{f}(E,t_{D};\tau )\rangle ,$$ (4)

where $\hat{\Theta }(R_{b}-R)$ is 1 for *R* < *R_b_* and 0; otherwise, *R_b_* is the interatomic distance after which all potential energy curves become dissociative, and *t_D_* is the time of arrival at the detector.

In propagating $\left|\Phi_{f}(E,t;\tau )\right\rangle$, which is later on used to evaluate Ω(*τ*), we use a Gaussian IR pulse of 800 nm central wavelength and 42.4 fs full-width at half-maximum of the intensity with a peak optical intensity *I*_0_ of 5.62 × 10^13^ W cm^−2^. The delay time between the pump and the probe pulse is varied from −100 fs up to 1500 fs in intervals of 0.5 fs each. This dense delay-time sampling is necessary to avoid aliasing effects when performing the Fourier transform due to the highly oscillatory ${N_{2}}^{2+}$ yield with respect to the delay time (cf. Fig. 3). Each wave-packet propagation for a particular delay time and Auger energy is carried out up to a final simulation time *t_F_* of 1700.0 fs. As for the pump pulse, we assume a Gaussian pulse of 5.0 fs duration.

The initial wave packets and the time-dependent basis function description to solve Eqs. (1)–(3) are taken to be the same as in Ref. 16. The propagation of $\left|\Phi_{f}(E,t;\tau )\right\rangle$ and the evaluation of Ω(*τ*) using Eq. (4) involve 3201 delay times, and for each delay time, the Auger energy is discretized into 24 energy steps.^16^ This results in 76 824 separate wave-packet propagations that need to be carried out.

In the actual pump-probe experiments, the atomic ions N^+^ and the remaining molecular dication ${N_{2}}^{2+}$ after the probe pulse are detected using an ion time-of-flight device with a time-of-flight on the order of *μ*s.^1^ Hence, the actual *t_D_* is orders of magnitude larger than the final wave-packet propagation time *t_F_*. This means that the possibility that the vibrational wave packets tunnel out of the quasi-bound potential energy curves after *t_F_* has to be considered. Tunneling corrections were calculated using the method developed by Le Roy and Bernstein,^30,31^ which is based on the WKB approximation. For a given quasi-bound vibrational energy *E_ν_* in electronic state *f*, the tunneling probability per collision against the dissociation energy barrier is, in atomic units

$$P_{f}(E_{\nu })=\text{exp}\;\left\{-2\int_{R_{2}(E_{\nu })}^{R_{3}(E_{\nu })}dR\;\sqrt{2\mu [V_{f}(R)-E_{\nu }]}\right\},$$ (5)

where *μ* is the reduced mass of the system and *V_f_*(*R*) is the potential energy curve of the corresponding electronic state. The integral in Eq. (5) is carried out between the second classical turning point and the exit point along the tunneling region below the potential energy barrier [*R*_2_(*E_ν_*), *R*_3_(*E_ν_*)]. On the other hand, the vibrational angular frequency is

$$\omega_{f}(E_{\nu })=2\pi {\left(\int_{R_{1}(E_{\nu })}^{R_{2}(E_{\nu })}dR\;\sqrt{\frac{2\mu }{E_{\nu }-V_{f}(R)}}\right)}^{-1},$$ (6)

where the integral is performed in the classical region [*R*_1_(*E_ν_*), *R*_2_(*E_ν_*)] between the first two classical turning points. The tunneling rate *k_f_*(*E_ν_*) is then given by

$$k_{f}(E_{\nu })=\frac{\omega_{f}(E_{\nu })}{2\pi }P_{f}(E_{\nu }),$$ (7)

and the tunneling coefficient for vibrational state *ν* in electronic state *f* follows from first-order kinetics

$$\zeta_{f}(E_{\nu })=e^{-k_{f}(E_{\nu })\;t_{D}},$$ (8)

where *t_D_* was defined earlier as the time needed to reach the detector (the time-of-flight of the ion in question). For ${N_{2}}^{2+}\;t_{D}=3.5\;\mu s$ is considered after Ref. 1.

**FIG. 1.**
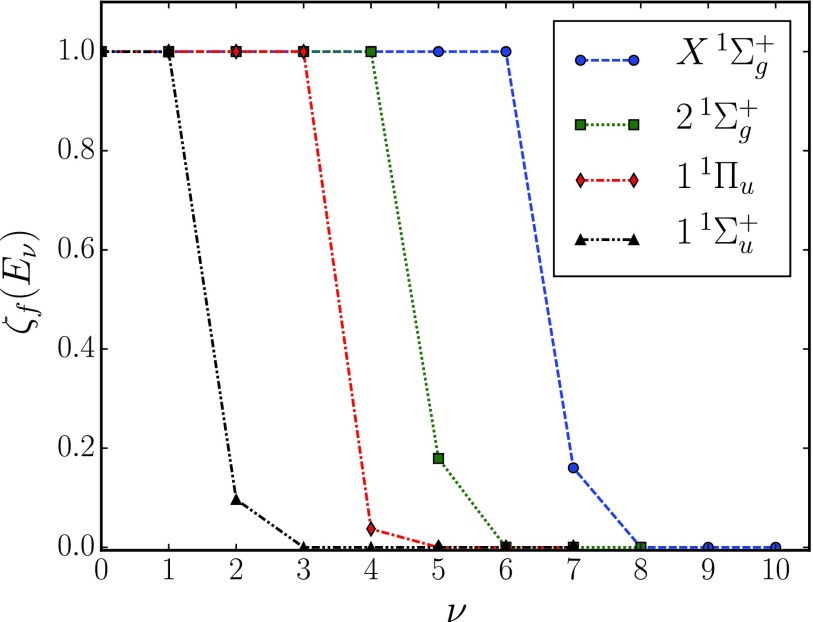
Tunneling coefficients of vibrational energy levels of the quasi-bound final electronic states of ${N_{2}}^{2+}$. These tunneling coefficients are calculated using Eq. (8) with *t_D_* = 3.5 *μ*s^1^ (see the text). The energy levels correspond to the vibrational eigenenergies presented in Table I.

The tunneling projector for each electronic state *f* is now defined as

$${\hat{P}}_{f}=\sum_{\nu }\zeta_{f}(E_{\nu })|\phi_{f}^{\nu }\rangle \langle \phi_{f}^{\nu }|,$$ (9)

where *ν* runs over all quasi-bound vibrational states in the final electronic state *f*. The total ${N_{2}}^{2+}$ yield as a function of delay time *τ* is now

$$\Omega (\tau )=\sum_{f}\int dE\;\langle \Phi_{f}(E,t_{F};\tau )|{\hat{P}}_{f}|\Phi_{f}(E,t_{F};\tau )\rangle ,$$ (10)

where Ω(*τ*) in Eq. (4) has been redefined to include the tunneling correction. The vibrational states for the tunneling correction are computed by artificially continuing the potential energy curves horizontally after the corresponding dissociation barrier. $\left|\phi_{f}^{\nu }\right\rangle$ in Eq. (9) corresponds to the *ν*-th vibrational eigenstate in such continued potentials. The corresponding eigenenergies are presented in Table I and their associated tunneling coefficients at the designated *t_D_* are shown in Fig. 1. The final propagation time *t_F_* is set 200 fs longer than the largest pump-probe delay time considered, such that all electronic populations have become stable after the IR pulse, and the newly promoted dissociative parts of the nuclear wave packets have been absorbed by a complex absorbing potential.^16^

**TABLE I.** Vibrational eigenenergies of the quasi-bound final electronic states of ${N_{2}}^{2+}$. The vibrational eigenstates and energies are calculated on modified potential energy curves continued horizontally from the top of the corresponding dissociation barrier onwards. The vibrational eigenenergies are given in THz and relative to the local minimum of the quasi-bound state $X^{1}\Sigma_{g}^{+}$.

Ν	$X^{1}\Sigma_{g}^{+}$	$2^{1}\Sigma_{g}^{+}$	1^1^Π_u_	$1^{1}\Sigma_{u}^{+}$
0	29.1	464.4	437.7	2057.3
1	86.4	515.8	477.6	2111.5
2	142.0	565.3	515.7	2164.6
3	195.7	612.4	551.9	2216.6
4	246.7	657.3	586.1	2267.3
5	293.7	699.6	617.9	2316.3
6	332.6	739.2	646.9	2363.4
7	359.8	775.5	671.9	2407.6
8	382.3	807.3		
9	402.3			
10	419.2			

### B. Perturbative analysis

As described in the previous section, the computational cost of the pump-probe signal is high due to the many propagations involved. Thus, we introduce a perturbative approximation of the effect of the IR probe on the ${N_{2}}^{2+}$ yield to extract the dynamical information. After time *t*_0_, which corresponds to a sufficiently long time after Auger decay such that all direct fragmentation has been completed (neglecting the tunneling effects) leaving only the quasi-bound ${N_{2}}^{2+}$, Eq. (3) is reduced to

$$i\frac{\partial }{\partial t}|\Phi_{f}(E,t;\tau )\rangle =({\hat{H}}_{f}+E)|\Phi_{f}(E,t;\tau )\rangle +\sum_{g\neq f}{\hat{\mu }}_{\mathrm{fg}}\cdot e_{\text{IR}}\varepsilon (t-\tau )|\Phi_{g}(E,t;\tau )\rangle .$$ (11)

In the interaction picture

$$\left|\Phi_{f}(E,t;\tau )\rangle =e^{-i({\hat{H}}_{f}+E)(t-t_{0})}{|}^{I}\Phi_{f}(E,t;\tau )\right\rangle .$$ (12)

Equation (11) reads

$$i\frac{\partial }{\partial t}{|}^{I}\Phi_{f}(E,t;\tau )\rangle =\sum_{g\neq f}e^{i({\hat{H}}_{f}+E)(t-t_{0})}{\hat{\mu }}_{\mathrm{fg}}\cdot e_{\text{IR}}\varepsilon (t-\tau )e^{-i({\hat{H}}_{g}+E)(t-t_{0})}{|}^{I}\Phi_{g}(E,t;\tau )\rangle ,$$ (13)

after some algebraic manipulations to simplify the expression. After going back to the Schrödinger picture, the first-order perturbation wave-function approximation to Eq. (13) is

$$|{\tilde{\Phi }}_{f}(E,t;\tau )\rangle =|\Phi_{f}^{\left(0\right)}(E,t)\rangle -i\sum_{g\neq f}\int_{t_{0}}^{t}dt\prime \;e^{-i({\hat{H}}_{f}+E)(t-t^{\prime })}{\hat{\mu }}_{\mathrm{fg}}\cdot e_{\text{IR}}\varepsilon (t\prime -\tau )|\Phi_{g}^{\left(0\right)}(E,t\prime )\rangle ,$$ (14)

where $\left|\Phi_{f}^{\left(0\right)}(E,t)\right\rangle$ is the unperturbed wave-function solution, i.e., the field-free wave function. We note that $\left|{\tilde{\Phi }}_{f}(E,t;\tau )\right\rangle$ may contain contributions from all electronic states *g* after the perturbative interaction with the probe pulse.

To simplify Eq. (14), we introduce an IR pulse with a broad bandwidth that covers all possible transitions and a duration much shorter than the vibrational periods on the quasi-bound potential energy curves, namely, a *δ*-pulse *ε*(*t*) = *ε*_0_ *δ*(*t*). For *t* > *τ*, this results in

$$|{\tilde{\Phi }}_{f}(E,t;\tau )\rangle =|\Phi_{f}^{\left(0\right)}(E,t)\rangle -i\varepsilon_{0}\sum_{g\neq f}e^{-i({\hat{H}}_{f}+E)(t-\tau )}{\hat{\mu }}_{\mathrm{fg}}\cdot e_{\text{IR}}|\Phi_{g}^{\left(0\right)}(E,\tau )\rangle .$$ (15)

Using the wave-function approximation in Eq. (15) and neglecting the tunneling correction, the approximated observable can be calculated as

$$\tilde{\Omega }(\tau )=\Omega^{\left(0,0\right)}(\tau )+\Omega^{\left(0,1\right)}(\tau )+\Omega^{\left(1,0\right)}(\tau )+\Omega^{\left(1,1\right)}(\tau ),$$ (16)

where the double-numbered superscripts refer to the respective perturbation order of the bras and kets plugged into Eq. (4). Specifically, these terms are

$$\Omega^{\left(0,0\right)}(\tau )=\sum_{f}\int dE\;\langle \Phi_{f}^{\left(0\right)}(E,t_{F})|\hat{\Theta }(R_{b}-R)|\Phi_{f}^{\left(0\right)}(E,t_{F})\rangle ,$$ (17)

$$\begin{array}{c} \Omega^{\left(0,1\right)}(\tau )+\Omega^{\left(1,0\right)}(\tau )=2\varepsilon_{0}\sum_{f}\sum_{g\neq f}\int dE\;ℑ\{\left\langle \Phi_{f}^{\left(0\right)}(E,t_{F})|\hat{\Theta }(R_{b}-R\right)\\ \times e^{-i({\hat{H}}_{f}+E)(t_{F}-\tau )}{\hat{\mu }}_{\mathrm{fg}}\cdot e_{\text{IR}}|\Phi_{g}^{\left(0\right)}(E,\tau )\rangle \}, \end{array}$$ (18)

$$\begin{array}{c} \Omega^{\left(1,1\right)}(\tau )=\varepsilon_{0}^{2}\sum_{f}\sum_{g\neq f}\sum_{g\prime \neq f}\int dE\;\langle \Phi_{g}^{\left(0\right)}(E,\tau )|{\hat{\mu }}_{\mathrm{gf}}\cdot e_{\text{IR}}e^{i({\hat{H}}_{f}+E)(t_{F}-\tau )}\hat{\Theta }(R_{b}-R)\\ \times e^{-i({\hat{H}}_{f}+E)(t_{F}-\tau )}{\hat{\mu }}_{\mathrm{fg}\prime }\cdot e_{\text{IR}}|\Phi_{g\prime }^{\left(0\right)}(E,\tau )\rangle . \end{array}$$ (19)

As can be appreciated in Eq. (17), the (0, 0) term is delay-independent. At *t* > *t*_0_, the only remaining wave-function amplitudes within the unfragmented region are from quasi-bound final states. Since $\tilde{\Omega }(\tau )$ concerns only the unfragmented wave packets at *t_F_*, we assume that the ${N_{2}}^{2+}$ yield from a pulse-induced transition remains unfragmented only when the transition is between quasi-bound states, i.e., $\hat{\Theta }(R_{b}-R)=\hat{1}$ when *f*, *g*, and g′ are quasi-bound electronic states, and 0 otherwise. By applying this assumption to Eq. (18), the time-evolution operator can act to the left resulting in a transition dipole-coupled term between $\left|\Phi_{f}^{\left(0\right)}(E,\tau )\right\rangle$ and $\left|\Phi_{g}^{\left(0\right)}(E,\tau )\right\rangle$, and its imaginary part cancels that of its complex conjugate, which also appears in the double sum in Eq. (18). Thus, the cross-term $\Omega^{\left(0,1\right)}(\tau )+\Omega^{\left(1,0\right)}(\tau )$ vanishes. By applying this assumption to Eq. (19), the time-evolution operators cancel each other and $\tilde{\Omega }(\tau )$ in Eq. (16) is simplified to

$$\tilde{\Omega }(\tau )=\Omega^{\left(0,0\right)}+\varepsilon_{0}^{2}\sum_{f}\sum_{g\neq f}\sum_{g\prime \neq f}\int dE\;\langle \Phi_{g}^{\left(0\right)}(E,\tau )|({\hat{\mu }}_{\mathrm{gf}}\cdot e_{\text{IR}})({\hat{\mu }}_{\mathrm{fg}\prime }\cdot e_{\text{IR}})|\Phi_{g^{\prime }}^{\left(0\right)}(E,\tau )\rangle .$$ (20)

To calculate $\tilde{\Omega }(\tau )$, field-free nuclear wave packets were propagated for *τ* up to 4000.0 fs using 0.5 fs intervals. The convolution integral with the x-ray pulse envelope and the Fourier transform of the resulting signal were then performed for *τ* > *t*_0_, where we set *t*_0_ = 200 fs.

## III. RESULTS AND DISCUSSION

### A. Perturbative analysis

**FIG. 2.**
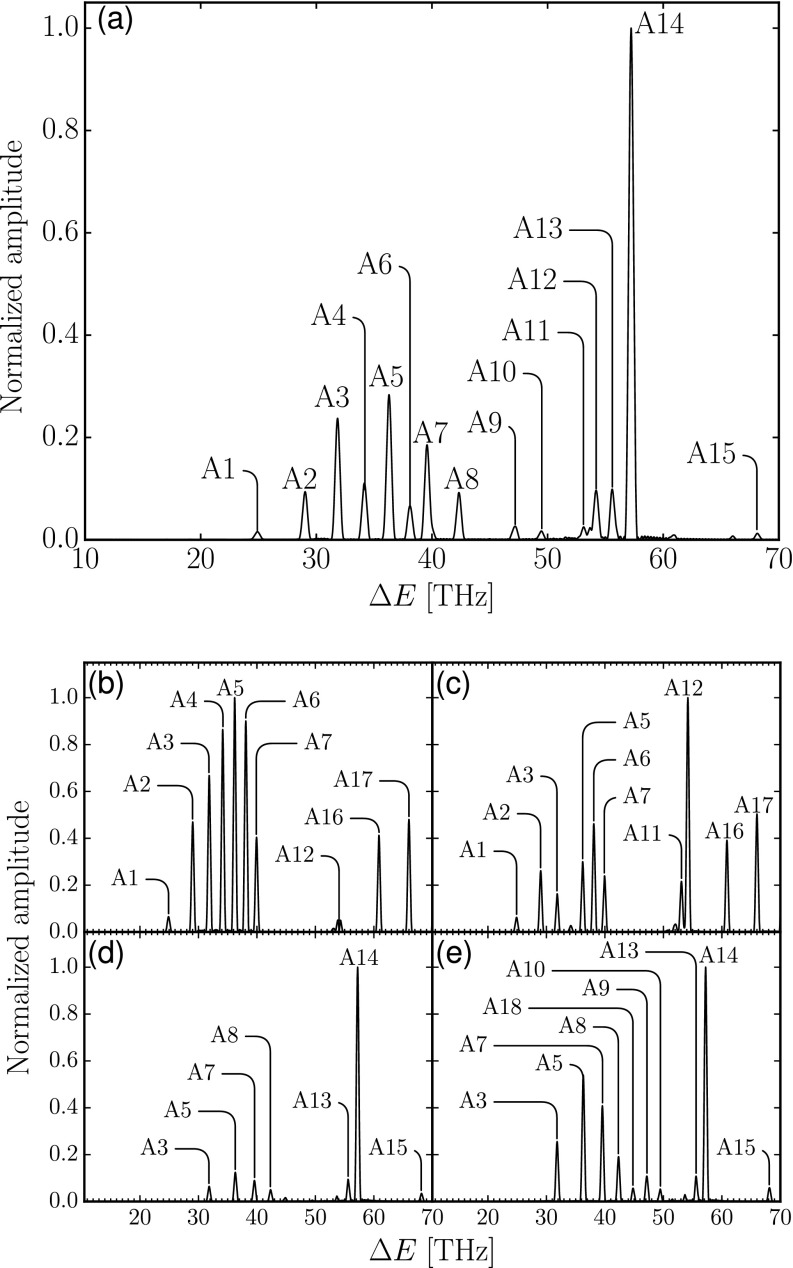
Spectra corresponding to the Fourier transform of the ${N_{2}}^{2+}$ yield $\tilde{\Omega }(\tau )$ obtained with the perturbative model for: (a) total yield in Eq. (20), and final quasi-bound states: (b) $X^{1}\Sigma_{g}^{+}$, (c) $2^{1}\Sigma_{g}^{+}$, (d) 1^1^Π_*u*_, and (e) $1^{1}\Sigma_{u}^{+}$ in Eq. (21). The frequencies in the spectra correspond to energy differences between vibrational quasi-bound states of ${N_{2}}^{2+}$. These peaks and the associated states are listed in Table II as well as their comparison with experiments.^1,4^

We first present the results of the perturbative analysis. Figure 2(a) and Table II show the Fourier spectrum and peaks of $\tilde{\Omega }(\tau )$ from the perturbative model in Eq. (20). Moreover, $\tilde{\Omega }(\tau )$ can also be decomposed into the contributions from each quasi-bound state *f*

$${\tilde{\Omega }}_{f}(\tau )=\varepsilon_{0}^{2}\sum_{g\neq f}\sum_{g\prime \neq f}\int dE\;\langle \Phi_{g}^{\left(0\right)}(E,\tau )|({\hat{\mu }}_{\mathrm{gf}}\cdot e_{\text{IR}})({\hat{\mu }}_{\mathrm{fg}\prime }\cdot e_{\text{IR}})|\Phi_{g\prime }^{\left(0\right)}(E,\tau )\rangle .$$ (21)

The Fourier peaks of ${\tilde{\Omega }}_{f}(\tau )$ for each quasi-bound final electronic state are shown in Figs. 2(b)–2(e). Upon inspection of terms in Eq. (21), one can show that the extracted frequencies are, in fact, energy differences between vibrational states of ${N_{2}}^{2+}$ (cf. Table I). To indicate the two vibrational levels of a quasi-bound electronic state involved in a given vibrational energy difference, we use the notation (*ν*_1_, *ν*_2_). For g′=g, each term in Eq. (21) is a combination of expansion coefficients of the involved vibrational states, transition dipole assisted coupling, and oscillating functions corresponding to vibrational energy differences *within* a quasi-bound electronic state. Meanwhile, for g′≠g, the vibrational states involved are from two different quasi-bound electronic states associated with the same final state *f* when interacting with the probe pulse.

**TABLE II.** Comparison of experimental^1,4^ and theoretical (this work) frequencies corresponding to vibrational energy differences in the final dicationic states. The two vibrational eigenstates involved are indicated in parenthesis. The uncertainty of the experimental data is 1.4 THz.^4^

Peak	Frequency (THz)		States
	This work	Expt.^1,4^	
A1	24.9		1^1^Π_*u*_ (6, 7)
A2	29.0	27.7	1^1^Π_*u*_ (5, 6)
A3	31.8	32.1	$2^{1}\Sigma_{g}^{+}\;\;(7,8),\;\;1^{1}\Pi_{u}\;\;(4,5)$
A4	34.2		1^1^Π_*u*_ (3, 4)
A5	36.2, 36.3	36.5	1^1^Π_*u*_ (2, 3), $2^{1}\Sigma_{g}^{+}\;(6,7)$
A6	38.1		1^1^Π_*u*_ (1, 2)
A7	39.6, 39.9		$2^{1}\Sigma_{g}^{+}\;\;(5,6),\;\;1^{1}\Pi_{u}\;\;(0,1)$
A8	42.3	42.3	$2^{1}\Sigma_{g}^{+}\;(4,5)$
A9	47.2		$2^{1}\Sigma_{g}^{+}\;(2,3)$
A10	49.5	49.6	$2^{1}\Sigma_{g}^{+}\;(1,2)$
A11	53.1		$1^{1}\Sigma_{u}^{+}\;(1,2)$
A12	54.2		$1^{1}\Sigma_{u}^{+}\;(0,1)$
A13	55.6		$X^{1}\Sigma_{g}^{+}\;(1,2)$
A14	57.2		$X^{1}\Sigma_{g}^{+}\;(0,1)$
A15	68.1		$2^{1}\Sigma_{g}^{+}\;(6,8)$
A16	60.9	59.8	1^1^Π_*u*_ (4, 6)
A17	66.0	65.7	1^1^Π_*u*_ (3, 5)
A18	44.8		$2^{1}\Sigma_{g}^{+}\;(3,4)$

As one can see from Eq. (21), for $f=X^{1}\Sigma_{g}^{+}$ and $2^{1}\Sigma_{g}^{+}$, the Fourier peaks shown in Figs. 2(b) and 2(c) originate from wave-packet oscillations in 1^1^Π_*u*_ and $1^{1}\Sigma_{u}^{+}$ states, which are coupled to the former states by transition dipole moments. Thus, these two spectra are similar in frequency, whereas the discrepancies in amplitude are from the transition dipole moments involved in those two states (cf. Fig. 3 of Ref. 16). The trend in peaks A1–A7 of Fig. 2(b) indicates that the population of middle-lying vibrational states in 1^1^Π_*u*_ is higher than for the corresponding few lowest and highest vibrational states. Apart from these two electronic states, peaks A3, A5, and A7 also appear in the spectra of 1^1^Π_*u*_ and $1^{1}\Sigma_{u}^{+}$ with slightly different frequencies [cf. Figs. 2(d), 2(e), and Table II]. These multiple peak assignments explain why the corresponding peaks in the spectrum of the total yield in Fig. 2(a) are higher than neighboring ones. Peaks A11 and A12 shown in Fig. 2(c) indicate that $1^{1}\Sigma_{u}^{+}$ has predominant population in the corresponding few lowest energy vibrational eigenstates. The g′≠g terms between 1^1^Π_*u*_ and $1^{1}\Sigma_{u}^{+}$ states, which contribute to higher frequencies, are washed out by the finite duration of the x-ray pulse.

Meanwhile, the sources of the Fourier peaks for 1^1^Π_*u*_ and $1^{1}\Sigma_{u}^{+}$ states shown in Figs. 2(d) and 2(e) are the wave-packet oscillations in $X^{1}\Sigma_{g}^{+}$ and $2^{1}\Sigma_{g}^{+}$ states, and thus, their frequencies are similar. The trend in peaks A3, A5, A7–A10, and A18 in Figs. 2(d) and 2(e) indicates that the higher vibrational states of $2^{1}\Sigma_{g}^{+}$ are more populated than the corresponding lower states. On the other hand, peaks A13 and A14 show that the few lowest vibrational states of $X^{1}\Sigma_{g}^{+}$ are predominantly populated during the Auger decay. This combination of small population in high vibrational states of $X^{1}\Sigma_{g}^{+}$ and in low vibrational states of $2^{1}\Sigma_{g}^{+}$ has the effect that the contributions of g′≠g terms between these two states are unobserved.

The predominant population of the low-lying vibrational states of $X^{1}\Sigma_{g}^{+}$ and $1^{1}\Sigma_{u}^{+}$ is to be expected given that the Franck-Condon regions of those two electronic states overlap with those of neutral and core-ionized N_2_, whereas those of $2^{1}\Sigma_{g}^{+}$ and 1^1^Π_*u*_ are shifted to a longer interatomic distance.^16^ Consequently, $2^{1}\Sigma_{g}^{+}$ and 1^1^Π_*u*_ states contribute to the dissociation as reported by Iwayama *et al.*^17^

Since the contribution of the g′≠g terms from pairs of distinct quasi-bound final electronic states is unobserved, Eq. (20) implies that the Fourier peaks in Fig. 2 come from the varying transition dipole moment functions within the unfragmented interatomic distance *R_b_*, that is, within the well of the quasi-bound final electronic states. If the transition dipole moment functions were constants, then $\tilde{\Omega }(\tau )$ would be delay-independent and the Fourier peaks in Fig. 2 would be unobserved. Later, we will also find an effect of this kind in the pump-probe simulations in Sec. III B.

Despite this simple approach, the frequencies corresponding to the vibrational energy differences agree, within the experimental uncertainty, with the recent data analysis^4^ of the pump-probe experiments described in Ref. 1. Table II compares the experimental frequencies shown in Fig. 7 of the supplementary material of Ref. 4 to the frequencies corresponding to the vibrational energy differences in Fig. 2 for the frequency range 10–70 THz. Our results permit an assignment of each peak to the quasi-bound dicationic electronic states. However, since the frequencies are related to vibrational energy differences in each electronic state, which can be close to each other, there are multiple possible assignments to some of the experimental peaks. We note, however, that we consider only singlet dication electronic states,^16^ while it is possible that other electronic states (e.g., triplet states) may also contribute to the vibrational energy difference spectra.

Since we assumed in Eq. (15) the probe pulse to be represented by a delta function, more peaks appear in the Fourier spectrum of the ${N_{2}}^{2+}$ yield than would be expected for a more spectrally selective probe pulse (as used in experiment). The pump-probe simulations in the following subsection confirm this expectation.

### B. Pump-probe simulations

**FIG. 3.**
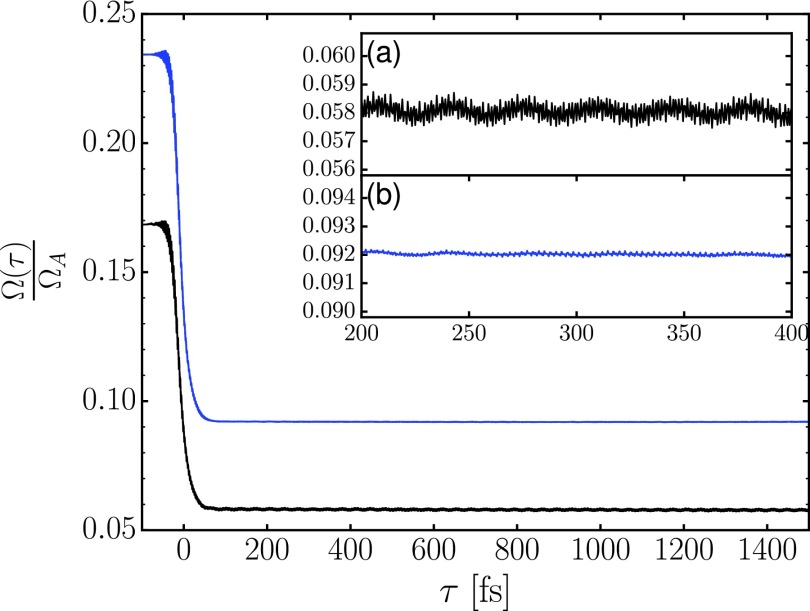
${N_{2}}^{2+}$ yield as a function of delay time with tunneling correction (lower black trace) and without tunneling correction (upper blue trace) before applying the convolution with the x-ray pulse envelope. The high frequency oscillation of the yield, which is shown in the inset panel (a) with and (b) without tunneling correction, is filtered out after the convolution. The slowly oscillating ${N_{2}}^{2+}$ yields reflect the dynamics of ${N_{2}}^{2+}$ in the quasi-bound final states.

Figure 3 shows the Auger-energy integrated ${N_{2}}^{2+}$ yield normalized by the total Auger yield

$$\Omega_{A}=\int dE\;\sum_{f}\underset{t\to \infty }{\lim}\langle \Phi_{f}(E,t)|\Phi_{f}(E,t)\rangle .$$ (22)

The ${N_{2}}^{2+}$ yield with tunneling correction is calculated using Eq. (10), while the one without tunneling correction is calculated using Eq. (4) evaluated at *t_D_* = *t_F_*. The summations in those two equations can also be decomposed into the contributions from each quasi-bound state *f* to obtain electronically resolved yield. At negative delay times, the IR pulse does not interact with the final states. For these negative delays, the blue curve indicates that, of all Auger decayed molecules, about 23% remain bound as ${N_{2}}^{2+}\;1700\;\text{fs}$ after the x-ray pulse. At the detector (after 3.5 *μ*s), the black curve, which includes the tunneling corrections, indicates a further decrease in the ${N_{2}}^{2+}$ yield to about 17% of the Auger yield Ω_*A*_. At positive delay times, the ${N_{2}}^{2+}$ yield is further reduced by the interaction with the IR probe pulse which promotes amplitude from quasi-bound to dissociative potential energy curves. This accounts for the further reduction in the ${N_{2}}^{2+}$ yield with and without tunneling correction by about 11% and 14% of the Auger yield Ω_*A*_, respectively. A large portion of this IR-induced dissociation at positive delay times arises from population transfer from $1^{1}\Sigma_{u}^{+}$ to 1^1^Π_*g*_. This is because the equilibrium distance of the former state matches the equilibrium distance of the N_2_ ground state and the ${N_{2}}^{+}$ core-ionized intermediate state. Furthermore, the energy spacing between the $1^{1}\Sigma_{u}^{+}$ and 1^1^Π_*g*_ states is close to resonant.^16^ The slope of the yield reduction in Fig. 3 around the pulse overlap region is steeper than that reported experimentally [cf. Fig. 5(c) of Ref. 1]. The difference is caused by the relatively large pump-probe timing jitter in the experiment.^1^

**FIG. 4.**
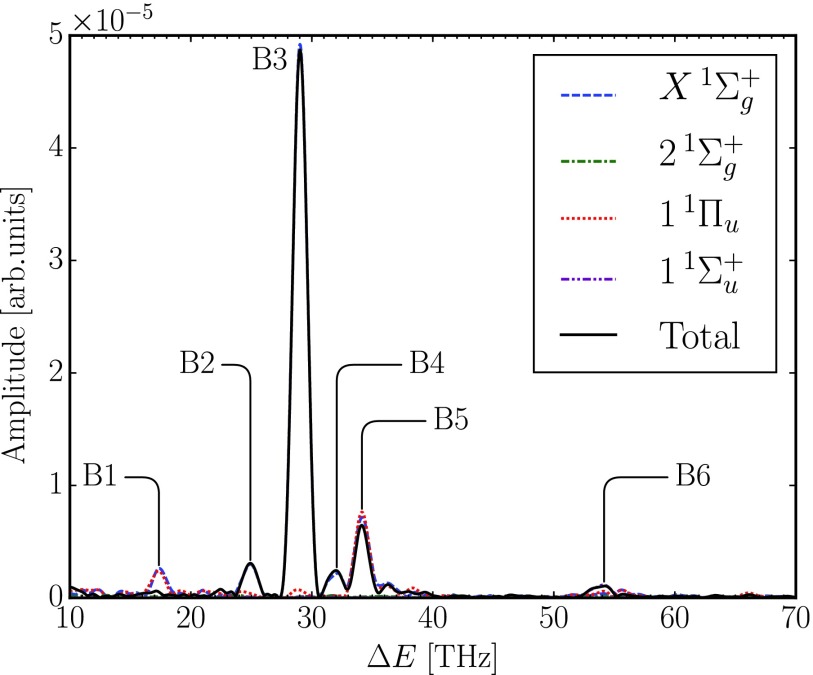
Total and electronically resolved Fourier spectra obtained from the full simulation of the ${N_{2}}^{2+}$ yield with tunneling correction. The electronically resolved spectra represent the contributions from the corresponding states to the oscillations of the ${N_{2}}^{2+}$ yield (see the text).

The signal of interest consists of the small modulations at delay times beyond 200 fs, for which the probe pulse interacts only with the metastable vibrational wave packets in the quasi-bound potential energy curves. The Fourier transform of the pump-probe delay signal, the ${N_{2}}^{2+}$ yield, was obtained similar to that in the perturbative model. The raw signal is first convolved with the x-ray pulse envelope and the Fourier transform is taken for delay times *τ* ≥ 200.0 fs. Note that now the IR probe pulse is already included in each single time-propagation. The Fourier spectra of the ${N_{2}}^{2+}$ yields with tunneling correction for each final quasi-bound electronic state as well as for the total yield are shown in Fig. 4. The peaks of the spectra are summarized in Table III.

**TABLE III.** Frequency peaks of the Fourier spectra of the pump-probe simulations of Fig. 4.

Peak	Frequency (THz)					Remark^a^
	$X^{1}\Sigma_{g}^{+}$	$2^{1}\Sigma_{g}^{+}$	1^1^Π_*u*_	$1^{1}\Sigma_{u}^{+}$	Total	
B1	17.4	…	17.3	…	…	(a)
	17.4	…	17.4	…	…	(b)
B2	24.9	…	…	…	24.9	(a)
	24.9	…	25.0	…	24.9	(b)
B3	29.0	…	…	…	29.0	(a)
	29.0	…	29.0	…	29.0	(b)
B4	32.1	…	…	…	32.0	(a)
	32.1	…	31.8	…	31.9	(b)
B5	34.2	…	34.1	…	34.1	(a)
	34.2	…	34.2	…	34.2	(b)
B6	…	…	…	54.1	54.2	(a)
	…	…	…	54.1	54.2	(b)

^a^ (a) Tunneling correction, (b) no tunneling correction.

Peaks of the total Fourier spectrum shown in Fig. 4 indicate that the unfragmented ${N_{2}}^{2+}$ population reaching the detector is oscillating at those frequencies as the delay time advances. This oscillation in population is dominated by the frequency of peak B3 with the second major contribution from peak B5. There are also small contributions coming from peaks B2, B4, and B6, while peak B1 does not appear in the total spectrum. By resolving the detected ${N_{2}}^{2+}$ yield electronically, i.e., decomposing the summation in Eq. (10) into the contribution from each quasi-bound state *f*

$$\Omega_{f}(\tau )=\int dE\;\langle \Phi_{f}(E,t_{F};\tau )|{\hat{P}}_{f}|\Phi_{f}(E,t_{F};\tau )\rangle$$ (23)

and obtaining spectra from each Ω_*f*_(*τ*), we can see that peaks B2, B3, and B4 are from the $X^{1}\Sigma_{g}^{+}$ state, while peaks B5 and B6 originate from the 1^1^Π_*u*_ and $1^{1}\Sigma_{u}^{+}$ states, respectively. The electronically resolved spectra also show that the contributions from the $X^{1}\Sigma_{g}^{+}$ and 1^1^Π_*u*_ states cancel each other out in peak B1 because the population transferred by the IR pulse among the two electronic states remains bound in this case, leading to no modulation of the total ${N_{2}}^{2+}$ yield.

Comparison of the A peaks in Table II and B peaks in Table III shows that only beating frequencies originating from 1^1^Π_*u*_ and $1^{1}\Sigma_{u}^{+}$ states are observed in the spectra of the numerical pump-probe simulations, whereas $X^{1}\Sigma_{g}^{+}$ and $2^{1}\Sigma_{g}^{+}$ states do not contribute to the B peaks. Since one of the major differences between the perturbative model giving rise to the A peaks and the numerical pump-probe simulations is the *δ*-pulse assumption [cf. Eq. (15)], these discrepancies between A and B peaks must also be related to this assumption. As has been noted earlier, since the use of this broad bandwidth limit of the IR pulse allows any possible transition, peaks from any vibrational energy differences with significant population and coupling function can be extracted. On the other hand, the use of an IR pulse of a certain duration, like the one used in the numerical pump-probe simulations, allows transitions only within its bandwidth, thus limiting the peaks that can be extracted from the ${N_{2}}^{2+}$ yield, and only a few frequencies in the A peaks in Table II appear in the B peaks in Table III. Moreover, the purely dissociative potential energy curves (cf. Fig. 2 of Ref. 16) are of *g* symmetry. Thus, within one-photon transitions, the quasi-bound electronic states that contribute to the B peaks are mostly of *u* symmetry. Nonetheless, $X^{1}\Sigma_{g}^{+}$, which is quasi-bound, contributes as well to modulations to the total ${N_{2}}^{2+}$ yield through coupling to *u* symmetry potential energy surfaces, as seen for example for peak B3 below. To further understand these phenomena, in the following, we discuss the origins of B peaks from the ${N_{2}}^{2+}$ yield before the tunneling dissociation takes place in an electronically and vibrationally resolved manner.

**FIG. 5.**
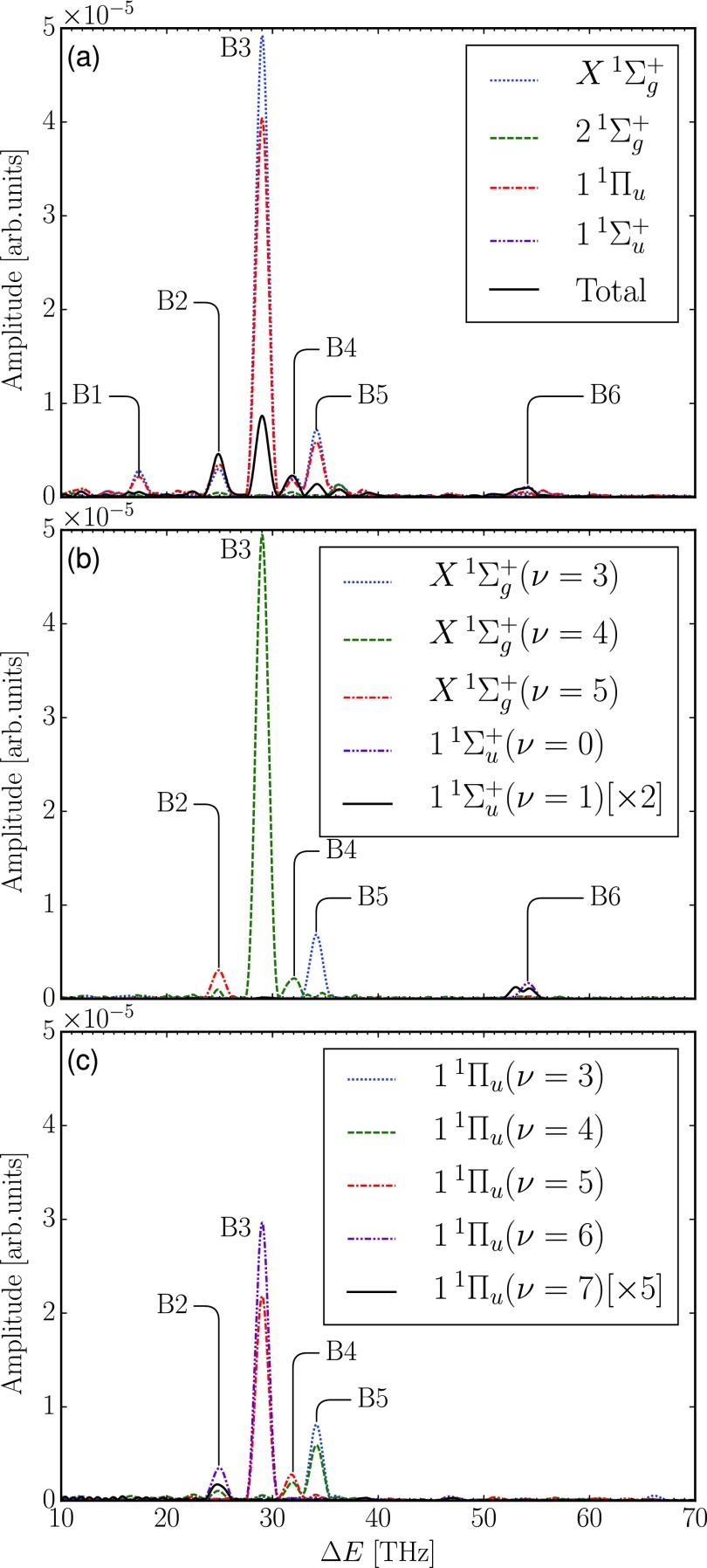
Fourier spectra obtained from the full simulation of the ${N_{2}}^{2+}$ yield without tunneling correction: (a) total and electronically resolved spectra and (b) and (c) vibrationally resolved spectra of $X^{1}\Sigma_{g}^{+},\;\;1^{1}\Sigma_{u}^{+}$, and 1^1^Π_*u*_ final states. The spectra show the contributions from the corresponding electronic and vibrational states to the oscillations of the ${N_{2}}^{2+}$ yield before the tunneling dissociation takes place (see the text).

The total and electronically resolved spectra without tunneling correction shown in Fig. 5(a) can be obtained from the ${N_{2}}^{2+}$ yields using the same equations as those with tunneling correction, i.e., Eqs. (10) and (23), respectively, by setting the tunneling coefficients *ζ_f_*(*E_ν_*) = 1. Furthermore, the vibrationally resolved spectra without tunneling correction shown in Figs. 5(b) and 5(c) can be obtained from the vibrationally resolved yields

$$\Omega_{f}^{\nu }(\tau )=\int dE\;\langle \Phi_{f}(E,t_{F};\tau )|\phi_{f}^{\nu }\rangle \langle \phi_{f}^{\nu }|\Phi_{f}(E,t_{F};\tau )\rangle ,$$ (24)

evaluated at the final simulation time *t_F_*, that is, prior to the tunneling dissociation.

As shown in Fig. 5, the most prominent peak B3 at 29.0 THz corresponds to the vibrational energy difference of 1^1^Π_*u*_ (5, 6) (cf. Fig. 2 and Table II). Unlike the broad bandwidth limit in the perturbative model, the 800 nm Gaussian IR pulse with limited bandwidth in these pump-probe simulations couples these vibrational states of 1^1^Π_*u*_ (*ν* = 5) and 1^1^Π_*u*_ (*ν* = 6), which oscillate at a beating frequency of 29.0 THz, to the dissociative 1^1^Π_*g*_ state more effectively at *R* close to the outer turning point of the quasi-bound potential well. There, the energy gap is close to resonant. Moreover, these two vibrational states are also resonantly coupled to the $X^{1}\Sigma_{g}^{+}\;(\nu =4)$ state by the probe pulse. From the previous discussion in Sec. III A, we know that the vibrational states 1^1^Π_*u*_ (*ν* = 5) and 1^1^Π_*u*_ (*ν* = 6) are initially populated. The incoming probe pulse at varying delay times modulates these two initially populated vibrational states and couples them to the dissociative 1^1^Π_*g*_ state and to the $X^{1}\Sigma_{g}^{+}\;(\nu =4)$ state. Thus, these vibrational states involved exhibit the same peak B3 at 29.0 THz as shown in Figs. 5(b) and 5(c). Moreover, since the population transfers between the states involved differ in phase, the total modulation in the ${N_{2}}^{2+}$ yield is smaller than the corresponding peak evaluated for each electronic state separately.

A fairly similar mechanism occurs as well for peak B2 shown in Fig. 5. In peak B2, the initially populated 1^1^Π_*u*_ (*ν* = 6) state and a much less populated 1^1^Π_*u*_ (*ν* = 7) state (as discussed in Sec. III A and shown in Fig. 2), which together oscillate at a frequency of 24.9 THz, are coupled by the probe pulse to the dissociative 1^1^Π_*g*_ state and to $X^{1}\Sigma_{g}^{+}\;(\nu =5)$ state giving rise to the peak B2 in the spectra of the corresponding electronic and vibrational states. Due to low initial population of the 1^1^Π_*u*_ (*ν* = 7) state, however, the amplitude of modulation of peak B2 is consequently lower than that of peak B3 (cf. Fig. 5).

The beating frequency indicated by peak B4 originates from the vibrational energy difference of 1^1^Π_*u*_ (4, 5) [cf. Fig. 5(c)]. These two states, which are initially populated after the Auger decay, are coupled to $X^{1}\Sigma_{g}^{+}\;(\nu =4)$ by the probe pulse, as discussed previously, thus giving rise to the similar peak in the spectra of the corresponding electronic and vibrational states. Meanwhile, the vibrational states 1^1^Π_*u*_ (*ν* = 3) and 1^1^Π_*u*_ (*ν* = 4), which oscillate with the beating frequency indicated by peak B5, are coupled by the IR probe pulse to $X^{1}\Sigma_{g}^{+}\;(\nu =3)$ state [see Figs. 5(a) and 5(b)]. Apart from the IR-induced coupling to the quasi-bound vibrational states, these two pairs of vibrational states are also weakly coupled to the dissociative channels giving rise to the total spectrum shown in Fig. 5(a).

So far, we have seen how the oscillation mechanism originates from the shape of the potential energy curves of the states involved. For the $1^{1}\Sigma_{u}^{+}$ state, however, the main source of the oscillation in the delay time signal is its transition dipole moment to the dissociative 1^1^Π_*g*_ state. This is similar to that of the perturbation model in Sec. III A. Within the quasi-bound potential well of $1^{1}\Sigma_{u}^{+}$ (roughly between 1.0 Å and 1.3 Å), the transition dipole moment function that couples $1^{1}\Sigma_{u}^{+}$ to 1^1^Π_*g*_ exhibits a large slope and, hence, drastically changes as a function of *R* (see Figs. 2(b) and 3(b) of Ref. 16). When the IR pulse arrives, the wave packet on this electronic state experiences a greater coupling function when ⟨R⟩ of the wave packet is close to the inner turning point of the quasi-bound potential well than when its ⟨R⟩ is close to the outer turning point of the well. Therefore, its coupling function is also oscillating as the wave packet oscillates in the quasi-bound well when the varying IR probe interacts with and couples it to the dissociative 1^1^Π_*g*_ state. Since essentially only the three lowest vibrational states of $1^{1}\Sigma_{u}^{+}$ are populated (cf. Table II), its population is oscillating with the frequency of the vibrational energy difference of $1^{1}\Sigma_{u}^{+}\;(0,1)$ manifested in peak B6 with a small side peak at 53.1 THz [cf. Fig. 5(b)]. This is the origin of the peak B6.

If one compares the Fourier spectra of the full simulations with and without tunneling correction in Figs. 4 and 5(a), respectively, one finds that peaks B2, B3, and B4 disappear in the 1^1^Π_*u*_ final state and peak B5 changes in the tunneling-corrected spectra. During the flight towards the detector, the vibrational 1^1^Π_*u*_ states for *ν* = 4 and higher undergo dissociation by tunneling (cf. Fig. 1). This has the effect that the corresponding modulations of the ${N_{2}}^{2+}$ yield and, thus, the resulting Fourier peaks B2, B3, and B4 are absent in the 1^1^Π_*u*_ (*ν* ≥ 4) final state in the spectra with tunneling correction. Peak B5 in the total spectrum, however, is more visible in the tunneling-corrected case. This is because one component in peak B5, namely, 1^1^Π_*u*_ (*ν* = 4), undergoes tunneling dissociation during the flight to the detector and does not contribute to the signal, while 1^1^Π_*u*_ (*ν* = 3) and $X^{1}\Sigma_{g}^{+}\;(\nu =3)$ remain unfragmented.

As expected, not all peaks among those in the perturbative model appear in the spectra of the actual pump-probe simulations. The discrepancies between the former and the latter spectra can be associated with the *δ*-pulse assumption made in the perturbative model, as well as the inclusion of tunneling predissociation in the pump-probe simulations. In the perturbative model, any dipole allowed transition contributes to and appears in the spectra, whereas in the full pump-probe simulations, the IR-induced transitions only contribute partially to the spectra. The discrepancies can also be associated with the nonperturbative nature of the pump-probe simulations. Although the A peaks originate from the leading-order of the perturbative model [cf. Eq. (20)], the nonperturbative nature of the full pump-probe simulations which generate the B peaks may very well exhibit features incompatible with the perturbative approximation, including the existence of peak B1 in the partial spectra.

A comparison of our result with the analysis by Fung *et al.* (cf. Fig. 4 of the main article and Fig. 7 of the supplementary material of Ref. 4), which is presented in Table II, shows that two peaks can be assigned from the simulations reported here, namely, peaks B3 (29.0 THz) and B4 (32.1 THz). We note, however, that in our simulations, we underestimated the pulse energy and did not use the same pulse parameters as the experiment in Ref. 1. We also note that the actual IR pulse intensity in the experiment of Ref. 1 may have been higher than the nominal value by a factor of two, possibly even by a factor of four.^32^ The appearance of peaks originating mostly from quasi-bound electronic states of *u*-type symmetry indicates that at peak optical intensity *I*_0_, the IR-induced transitions in the pump-probe simulations arise from one-photon transitions and two-photon processes are mostly excluded. It cannot be ruled, however, that an increased peak optical intensity of the IR pulse beating frequencies involving *g*-type quasi-bound electronic states, which are partly observed in the data analysis, can be observed in the ${N_{2}}^{2+}$ yield via two-photon transitions to dissociative *g*-symmetry states from the ground state electronic state of the dication.

## IV. CONCLUSIONS

We report real-time wave-packet simulations of x-ray pump-IR probe experiments on molecular nitrogen. As it is well known, several electronic states of ${N_{2}}^{2+}$ are quasi-bound, namely, $X^{1}\Sigma_{g}^{+},\;2^{1}\Sigma_{g}^{+},\;1^{1}\Pi_{u}$, and $1^{1}\Sigma_{u}^{+}$.^16^ The N_2_ molecules are first core-ionized by the x-ray pulse and subsequently undergo Auger decay. The IR probe pulse then interrogates the nuclear wave-packet dynamics of ${N_{2}}^{2+}$ in the quasi-bound potential energy curves of the dicationic states mentioned above. This work is motivated by the first time-resolved pump-probe experiments at LCLS^1^ and the sophisticated data-analytical work that followed.^4^ We assume a Gaussian x-ray pump pulse of duration 5.0 fs and an IR probe pulse of 800 nm central wavelength and 42.4 fs duration. We also vary the peak optical intensity of the IR probe pulse to qualitatively determine its effect on the frequencies of the pump-probe spectrum.

On the basis of a perturbative treatment of the probe step, we first demonstrate that it is possible to extract dynamical information from the time-resolved ${N_{2}}^{2+}$ yield modulated by the delay time of the IR probe pulse. This perturbative treatment yields all frequencies participating in the vibrational wave packets that can potentially be revealed by the probe at a fraction of the computational cost of the complete simulations. The frequencies extracted from this treatment are in good agreement with the frequencies extracted from the experimental data.^4^

The complete simulations include the probe pulse explicitly as well as the tunneling corrections of the populations of metastable vibrational levels. The simulated spectrum shows three main peaks at 24.9, 29.0, and 34.1 THz with several lower peaks around them. These peaks are mainly due to population transfer among 1^1^Π_*u*_ and 1^1^Π_*g*_ states induced by the IR probe pulse at *R* close to the inner barrier turning points. The assignment of these peaks to vibrational energy differences between specific pairs of vibrational states in a particular electronic state is reported as well. Two common frequencies (29.0 and 32.1 THz) are found by comparison of the data-analytical and theoretical results. From the results of the peak optical intensity variation, we have an indication that there may have been some inaccuracy between the measured peak optical intensity and the one experienced by the ${N_{2}}^{2+}$ molecules in the experiment.^1^

## References

[1] J. M. Glownia , J. Cryan , J. Andreasson , A. Belkacem , N. Berrah , C. I. Blaga , C. Bostedt , J. Bozek , L. F. DiMauro , L. Fang , J. Frisch , O. Gessner , M. Gühr , J. Hajdu , M. P. Hertlein , M. Hoener , G. Huang , O. Kornilov , J. P. Marangos , A. M. March , B. K. McFarland , H. Merdji , V. S. Petrovic , C. Raman , D. Ray , D. A. Reis , M. Trigo , J. L. White , W. White , R. Wilcox , L. Young , R. N. Coffee , and P. H. Bucksbaum , Opt. Express 18, 17620 (2010). 10.1364/OE.18.017620 20721148

[2] S. K. Semenov , M. S. Schöffler , J. Titze , N. Petridis , T. Jahnke , K. Cole , L. P. H. Schmidt , A. Czasch , D. Akoury , O. Jagutzki , J. B. Williams , T. Osipov , S. Lee , M. H. Prior , A. Belkacem , A. L. Landers , H. Schmidt-Böcking , T. Weber , N. A. Cherepkov , and R. Dörner , Phys. Rev. A 81, 043426 (2010). 10.1103/PhysRevA.81.043426

[3] N. A. Cherepkov , S. K. Semenov , M. S. Schöffler , J. Titze , N. Petridis , T. Jahnke , K. Cole , L. P. H. Schmidt , A. Czasch , D. Akoury , O. Jagutzki , J. B. Williams , T. Osipov , S. Lee , M. H. Prior , A. Belkacem , A. L. Landers , H. Schmidt-Böcking , R. Dörner , and T. Weber , Phys. Rev. A 82, 023420 (2010). 10.1103/PhysRevA.82.023420

[4] R. Fung , A. M. Hanna , O. Vendrell , S. Ramakrishna , T. Seideman , R. Santra , and A. Ourmazd , Nature 532, 471 (2016). 10.1038/nature17627 27121840

[5] C. S. Lehmann , A. Picón , C. Bostedt , A. Rudenko , A. Marinelli , D. Moonshiram , T. Osipov , D. Rolles , N. Berrah , C. Bomme , M. Bucher , G. Doumy , B. Erk , K. R. Ferguson , T. Gorkhover , P. J. Ho , E. P. Kanter , B. Krässig , J. Krzywinski , A. A. Lutman , A. M. March , D. Ray , L. Young , S. T. Pratt , and S. H. Southworth , Phys. Rev. A 94, 013426 (2016). 10.1103/PhysRevA.94.013426

[6] W. Eberhardt , E. W. Plummer , I. W. Lyo , R. Carr , and W. K. Ford , Phys. Rev. Lett. 58, 207 (1987). 10.1103/PhysRevLett.58.207 10034870

[7] W. E. Moddeman , T. A. Carlson , M. O. Krause , B. P. Pullen , W. E. Bull , and G. K. Schweitzer , J. Chem. Phys. 55, 2317 (1971). 10.1063/1.1676411

[8] H. Ågren , J. Chem. Phys. 75, 1267 (1981). 10.1063/1.442176

[9] S. Svensson , A. N. de Brito , M. P. Keane , N. Correia , L. Karlsson , C.-M. Liegener , and H. Agren , J. Phys. B: At. Mol. Opt. Phys. 25, 135 (1992). 10.1088/0953-4075/25/1/017

[10] G. Víkor , S. Ricz , L. Tóth , B. Sulik , J. Végh , A. Kövér , and L. Kövér , Nucl. Instrum. Methods Phys. Res., Sect. B 124, 393 (1997). 10.1016/S0168-583X(96)00870-1

[11] S. L. Sorensen , C. Miron , R. Feifel , M. N. Piancastelli , O. Björneholm , and S. Svensson , Chem. Phys. Lett. 456, 1 (2008). 10.1016/j.cplett.2008.03.015

[12] J. P. Cryan , J. M. Glownia , J. Andreasson , A. Belkacem , N. Berrah , C. I. Blaga , C. Bostedt , J. Bozek , N. A. Cherepkov , L. F. DiMauro , L. Fang , O. Gessner , M. Gühr , J. Hajdu , M. P. Hertlein , M. Hoener , O. Kornilov , J. P. Marangos , A. M. March , B. K. McFarland , H. Merdji , M. Messerschmidt , V. S. Petrović , C. Raman , D. Ray , D. A. Reis , S. K. Semenov , M. Trigo , J. L. White , W. White , L. Young , P. H. Bucksbaum , and R. N. Coffee , J. Phys. B: At. Mol. Opt. Phys. 45, 055601 (2012). 10.1088/0953-4075/45/5/055601

[13] B. Kempgens , A. Kivimäki , M. Neeb , H. M. Köppe , A. M. Bradshaw , and J. Feldhaus , J. Phys. B: At. Mol. Opt. Phys. 29, 5389 (1996). 10.1088/0953-4075/29/22/016 10060650

[14] U. Hergenhahn , O. Kugeler , A. Rüdel , E. E. Rennie , and A. M. Bradshaw , J. Phys. Chem. A 105, 5704 (2001). 10.1021/jp0038456

[15] M. S. Schöffler , J. Titze , N. Petridis , T. Jahnke , K. Cole , L. P. H. Schmidt , A. Czasch , D. Akoury , O. Jagutzki , J. B. Williams , N. A. Cherepkov , S. K. Semenov , C. W. McCurdy , T. N. Rescigno , C. L. Cocke , T. Osipov , S. Lee , M. H. Prior , A. Belkacem , A. L. Landers , H. Schmidt-Böcking , T. Weber , and R. Dörner , Science 320, 920 (2008). 10.1126/science.1154989 18487190

[16] A. M. Hanna , O. Vendrell , A. Ourmazd , and R. Santra , Phys. Rev. A 95, 043419 (2017). 10.1103/PhysRevA.95.043419

[17] H. Iwayama , T. Kaneyasu , Y. Hikosaka , and E. Shigemasa , J. Chem. Phys. 145, 034305 (2016). 10.1063/1.4958620 27448885

[18] R. W. Wetmore and R. K. Boyd , J. Phys. Chem. 90, 5540 (1986). 10.1021/j100280a013

[19] P. R. Taylor and H. Partridge , J. Phys. Chem. 91, 6148 (1987). 10.1021/j100308a018

[20] F. R. Bennett , Chem. Phys. 190, 53 (1995). 10.1016/0301-0104(94)00348-E

[21] P. A. Martin , F. R. Bennett , and J. P. Maier , J. Chem. Phys. 100, 4766 (1994). 10.1063/1.466267

[22] M. Lundqvist , D. Edvardsson , P. Baltzer , and B. Wannberg , J. Phys. B: At. Mol. Opt. Phys. 29, 1489 (1996). 10.1088/0953-4075/29/8/013

[23] W. Jiang , Y. G. Khait , and M. R. Hoffmann , J. Chem. Phys. 127, 164308 (2007). 10.1063/1.2790439 17979339

[24] A. Pandey , B. Bapat , and K. R. Shamasundar , J. Chem. Phys. 140, 034319 (2014). 10.1063/1.4861665 25669391

[25] F. Fantuzzi , T. M. Cardozo , and M. A. C. Nascimento , Phys. Chem. Chem. Phys. 19, 19352 (2017). 10.1039/C7CP02792C 28703821

[26] H.-D. Meyer , G. A. Worth , M. H. Beck , A. Jäckle , M.-C. Heitz , S. Wefing , U. Manthe , S. Sukiasyan , A. Raab , M. Ehara , C. Cattarius , F. Gatti , F. Otto , M. Nest , A. Markmann , M. R. Brill , and O. Vendrell , “ The MCTDH Package, Version 8.4, 2007” (2007).

[27] M. H. Beck and H.-D. Meyer , Z. Phys. D: At., Mol. Clusters 42, 113 (1997). 10.1007/s004600050342

[28] M. H. Beck , A. Jäckle , G. A. Worth , and H. D. Meyer , Phys. Rep. 324, 1 (2000). 10.1016/S0370-1573(99)00047-2

[29] H.-D. Meyer and G. A. Worth , Theor. Chem. Acc. 109, 251 (2003). 10.1007/s00214-003-0439-1

[30] R. J. Le Roy and R. B. Bernstein , J. Chem. Phys. 54, 5114 (1971). 10.1063/1.1674805

[31] R. J. Le Roy and W. Liu , J. Chem. Phys. 69, 3622 (1978). 10.1063/1.437070

[32] R. N. Coffee , private communication (2018).

